# Screening for cardiovascular disease risk factors beginning in childhood

**DOI:** 10.1186/s40985-015-0011-2

**Published:** 2015-11-05

**Authors:** Clemens Bloetzer, Pascal Bovet, Joan-Carles Suris, Umberto Simeoni, Gilles Paradis, Arnaud Chiolero

**Affiliations:** 1 Department of Pediatrics, Lausanne University Hospital, Lausanne, Switzerland; 2 Division of Chronic Diseases, Institute of social and preventive medicine, Lausanne University Hospital, Lausanne, Switzerland; 3 Department of Epidemiology, Biostatistics, and Occupational Health, McGill University, Montreal, Canada

**Keywords:** Screening, Cardiovascular disease, Children, Adolescents

## Abstract

Cardiovascular diseases (CVD) are the leading cause of death worldwide. Individual detection and intervention on CVD risk factors and behaviors throughout childhood and adolescence has been advocated as a strategy to reduce CVD risk in adulthood. The U.S. National Heart, Lung, and Blood Institute (NHLBI) has recently recommended universal screening of several risk factors in children and adolescents, at odds with several recommendations of the U.S. Services Task Force and of the U.K. National Screening committee. In the current review, we discuss the goals of screening for CVD risk factors (elevated blood pressure, abnormal blood lipids, diabetes) and behaviors (smoking) in children and appraise critically various screening recommendations. Our review suggests that there is no compelling evidence to recommend universal screening for elevated blood pressure, abnormal blood lipids, abnormal blood glucose, or smoking in children and adolescents. Targeted screening of these risk factors could be useful but specific screening strategies have to be evaluated. Research is needed to identify target populations, screening frequency, intervention, and follow-up. Meanwhile, efforts should rather focus on the primordial prevention of CVD risk factors and at maintaining a lifelong ideal cardiovascular health through environmental, policy, and educational approaches.

## Introduction

Cardiovascular diseases (CVD) are the leading cause of death worldwide with a burden of over 17 million deaths per year (31 % of the global total) [1]. They are frequent in elderly but occur rarely before the age of 60 years. Nevertheless, the pathogenic process of atherosclerosis causing CVD begins early in life, in particular during childhood and adolescence [2, 3]. CVD risk factors and risk behaviors can be detected in childhood, and the extent of their presence has been linked to the severity of atherosclerosis in childhood and in adulthood [2–6]. There is also growing evidence that CVD risk factors and behaviors track to various degrees into adulthood, contributing to the risk of diseases decades later [7–11]. Therefore, individual detection and intervention on CVD risk factors and behaviors throughout childhood and adolescence has been advocated as a strategy to reduce CVD risk in adulthood [5, 6, 12].

Hence, in a viewpoint published in 2009, McGill et al. argued that “[…] pediatricians [should be] responsible for the prevention of adult cardiovascular diseases” [6]. Indeed, as the development of CVD begins early in life and CVD risk factors are identifiable in children, he argued that individualized clinical prevention of CVD beginning in childhood should be considered. Recent recommendations of the U.S. National Heart, Lung, and Blood Institute (NHLBI) for the universal screening of several risk factors are coherent with this approach and have raised a debate about the role of CVD risk factors screening during childhood [13].

In the current review, we discuss the goals of screening for several CVD risk factors (elevated blood pressure, abnormal blood lipids, diabetes) and behaviors (smoking) in children and adolescents (below 19 years of age) and appraise critically various screening recommendations.

## Review

### Goals and peculiarities of screening for CVD risk factors in children and adolescents

Possible strategies for CVD prevention starting in childhood are either to prevent the development of risk factors in the first place (primordial prevention) [12] or to identify and treat children with major risk factors predisposing them to develop clinical disease several decades later (primary prevention) [5, 6]. For the later strategy, the identification of children is possible through screening. The aim of any screening activity is to identify in a healthy population the individuals who are at increased risk for a disease or who have the disease at an early stage [14, 15]. Once screened, these individuals are offered further testing to confirm or not the presence of the disease and, if necessary, have early intervention either to cure the disease or to prevent major consequences of the disease [15]. In CVD prevention starting in childhood, screening aims therefore to detect children carrying risk factors or risk behaviors for CVD, in order to intervene early and reduce their risk to develop clinical manifest diseases later in life.

Following the classical Wilson-Jungner criteria to assess whether any condition potentially warrants screening efforts, several conditions have to be fulfilled before recommending screening for CVD risk factors in children (Table 1) [16, 17]. A first criteria is that the prevalence of the screened condition should be known. While prevalence of smoking in children and adolescents is relatively well documented in many populations [18], much less is known on the prevalence of elevated blood pressure [19–21] and dyslipidemia [22], and the situation is even worst for diabetes [23–25]. Second, the natural history linking risk factors to CVD and the absolute risk of CVD associated with a given level of risk factors should be documented. While this risk is well documented in adults, only indirect evidence exists in children, i.e., through cohort studies having shown the association between CVD risk factors in childhood and surrogate marker of CVD in young adulthood [2–6, 26, 27]. Third, a valid and reliable screening test should exist, different screening strategies should have been evaluated, and reference values (for blood pressure, lipids, and glucose) should be available [20, 22, 28]. Finally, there should be an agreement on whom to treat and the benefits, harms, and costs of treatment should be documented. As screening engages healthy children and adolescents and since the treatment may be necessary for many decades, the balance of benefits and harms is of particular concern. Avoidance of overdiagnosis and overtreatment is of major importance in the evaluation of screening strategies [29].

**Table 1:** Issues to consider when assessing the relevance of screening for cardiovascular disease (CVD) risk factors in children and adolescents. Adapted from the Wilson-Jungner criteria [17]

1. The condition should be a major and modifiable risk factor for CVD with a known prevalence in the population	
2. The absolute risk of CVD associated with a given level of risk factor should be known	
3. There should be a valid, reliable, and acceptable screening test to identify the risk factor in children and adolescent	
4. The best screening strategy should be known (e.g., universal versus targeted screening)	
5. There should be an agreement on whom to treat; further, the benefits harms, and costs of treatment should be known (treatment early in life should be associated with a better outcome than treatment later in life, accounting for lead time bias)	

### Recommendations for the screening for CVD risk factors in children and adolescents

In the integrated guidelines for the cardiovascular health, experts from the NHLBI recommend the screening of several CVD risk factors at various ages during childhood (Table 2) [13]. However, most of these recommendations are at odds with the recommendations of the U.S. Preventive Services Task Force (USPSTF) [30] and of the U.K. National Screening Committee [31].

**Table 2:** Screening of cardiovascular diseases (CVD) risk factors in children and adolescents recommended by the the U.S. National Heart, Lung, and Blood Institute (NHLBI) [13], by the U.S. Preventive Services Task Force (USPSTF) [30], and by the U.K. National Screening committee [31]

Screening	NHLBI recommendations	USPSTF recommendations	U.K. National Screening committee
Elevated blood pressure	Annual blood pressure measurement in all children from age 3 year; targeted measurement in infants with renal/urologic/cardiac diagnosis or history of neonatal intensive care before the age of 3 year	Evidence insufficient to assess the balance of benefits and harms of screening for primary hypertension in asymptomatic children and adolescents to prevent subsequent cardiovascular disease in childhood or adulthood.	Systematic population screening not recommended
Dyslipidemia/abnormal blood lipids	Universal lipid screening at age 9–11 year; measurement of non-fasting or fasting lipid profile; targeted screening according to family history or other high risk condition before the age of 9–11 year	Evidence insufficient to recommend for or against routine screening for lipid disorders in infants, children, adolescents, or young adults (up to age 20).	Systematic population screening not recommended
Diabetes	Targeted screening at age 9–11 year following the American Diabetes Association guidelines, i.e., in overweight children and with two or more additional risk factors for diabetes [ADA 2014]	No specific recommendation for children or adolescents	No specific recommendation for children or adolescents
Smoking	Assessment of tobacco use beginning at 9–11 year	Recommendation that primary care clinicians provide interventions, including education or brief counseling, to prevent initiation of tobacco use among school-aged children and adolescents. No recommendation to screen for tobacco use.	No specific recommendation for children or adolescents

#### Screening for elevated blood pressure

Hypertension is a major risk factor for CVD in adults [32]. As there is growing evidence that sustained elevated blood pressure in childhood causes persistent cardiovascular alterations and tracks into adulthood, several guidelines recommended universal screening for hypertension in childhood starting at the age of three [13, 33, 34]. However, recent, exhaustive reviews have concluded that the evidence to recommend universal hypertension screening in childhood was limited [21, 35, 36].

While it seems reasonable to assume that children with hypertension are at increased risk for CVD in adult life, no study has lasted long enough to link directly elevated blood pressure in childhood to the risk of CVD in adulthood. The absolute risk of CVD associated with a given pediatric blood pressure level is, indeed, unknown [20, 21]. As a consequence, a BP threshold above which intervention is beneficial is not known in children. Further, although lifestyle or pharmacologic interventions for hypertension in childhood have been shown to decrease blood pressure in the short-term, the long-term benefits and harm are unknown [20]. It is therefore unknown whether potential harms outweigh the expected benefices.

Due to the length of the pathogenic process and since CVD occurs rarely before the age of 60, one can assume that no randomized controlled trial to assess the direct effect of any BP reduction intervention in childhood on the absolute risk of CVD will be ever conducted. In a cost-effective simulation study, neither universal screening with treatment of those with hypertension or treatment of those with end-organ injury, nor targeted screening in overweight adolescents was shown to perform better than population-based interventions [37].

**Fig. 1:**
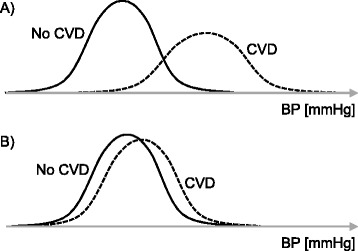
Blood pressure would be an ideal risk marker for screening if the distribution of blood pressure in individuals having a cardiovascular disease (CVD) was very different than in individuals not having a CVD (**a**). However, blood pressure is a relatively weak risk factor for CVD (i.e., elevated blood pressure is a poor discriminator for sorting out individuals who will have a CVD form other individuals) as there is no large difference in the distribution of blood pressure between individuals having CVD and individuals not having a CVD (**b**) [14, 38]. The same is true for blood lipids

A further major issue is that CVD risk factors like blood pressure or blood lipids are relatively weak CVD predictors (Fig. 1) [14, 38]. Indeed, the discriminative power of blood pressure or blood lipids for having or not a CVD later in life is weak. For instance, while individuals with elevated blood pressure have a higher risk of CVD, many cases of CVD will occur in individuals with normal blood pressure. This is why high risk prevention strategies based notably on screening are not sufficient to prevent CVD in the population, what requires population-based prevention strategies to have a favorable effect on the distribution of risk factors in the whole population [39, 40].

#### Screening for dyslipidemia

In adults, high levels of total cholesterol and low density lipoprotein-cholesterol (LDL-C) are important risk factors for coronary heart disease. Further, there is also good evidence that lipid-lowering treatment substantially decreases the incidence of coronary heart disease in persons with dyslipidemia [41]. Therefore, the USPSTF strongly recommends blood lipids screening in men aged 35 and women aged 45 [42]. It is also well known that the effect of lipid-lowering treatment will depend on the absolute risk of CVD. As age is the main determinant of CVD risk, the USPSTF does not recommend screening before the age of 35 in men and 45 in women as below that age, the absolute risk of CVD is low and the benefit of treatment is expected to be minimal. In younger adults, the USPSTF recommends targeted screening only in high risk individuals, i.e., having a family history of CVD at a young age, smokers, or a personal history of diabetes or hypertension [42].

In children, the USPSTF argue that evidence is insufficient to recommend for or against routine screening for lipid disorders in infants, children, adolescents, or young adults (up to age 20) [43] (an update of this recommendation is ongoing). The UK National Screening committee does not recommend screening in children. At odds with this recommendation, the NHLBI recommended in 2012 universal screening for dyslipidemia in children aged 9–11 years old and targeted screening in high risk children at younger age [13]. These recommendations have raised an important debate, with strong doubts about their efficiency and concerns about their potential harms [44–47].

Following the NHLBI guidelines, the primary goal of universal screening is identifying cases of familial hypercholesterolemia (FH) [46]. FH is indeed the most prevalent primary dyslipidemia in children, reaching 1 in 250–500 of individuals [48]. It is a severe form of dyslipidemia associated with a very high risk of CVD; the cumulative risk for coronary heart disease is estimated to be greater than 50 % in men by the age of 50 and 30 % in women by the age of 60 [49]. Statin treatment in these children is efficient (with beneficial effect shown on surrogates markers of CVD) and safe [46]. However, while lifetime treatment is potentially required, long term safety is not established.

Targeted screening to high risk children (e.g., with obesity or a family history of CVD at an early age) and cascade screening (i.e., systematic screening of close relatives of previously diagnosed index cases) has been proposed as alternative to universal screening for the detection of FH [48, 49]. For instance, the UK National Institute for Health and Clinical Excellence recommends cascade screening using a combination of genetic testing and LDL cholesterol concentration measurement of close (first-, second-, and third-degree) biological relatives of people with FH [49]. Pediatricians favor targeted screening as shown by the higher proportion of test for blood lipids among children with obesity in the United States [50].

#### Screening for diabetes

Hyperglycemia accelerates atherosclerosis and is a cause of CVD [51, 52]. Screening for abnormal blood glucose (impaired fasting glucose or impaired glucose tolerance) and type 2 (T2DM) diabetes mellitus is recommended by the USPSTF in adults at high risk of diabetes [53]. The goal of screening in adults is to prevent progression of abnormal blood glucose to T2DM and long term complications of diabetes, either microvascular (retinopathy, neuropathy, nephropathy) or macrovascular (i.e., CVD) complications. Early treatment of T2DM in adults has been shown to be associated with a lower rate of microvascular complications.

In children, type 1 (T1DM) diabetes mellitus is much more frequent than T2DM [23–25]. T1DM is a different condition than T2DM and characterized by a lack of insulin secretion by the pancreas. T1DM develops rapidly, sometimes with dramatic consequences, and there is no true preclinical indicator that could be easily targeted for screening. Due to the increasing prevalence of obesity, abnormal blood glucose and T2DM is expected to be increasingly frequent in children and adolescents [25, 54]. Elevated blood glucose in childhood is a predictor of prediabetes and T2DM in adulthood [54].

While there is no specific recommendation by the USPSTF or by the UK National Screening committee for the screening of abnormal blood glucose and T2DM, several guidelines recommend targeted screening for glucose intolerance in children at high risk, e.g., due to the presence of overweight or other risk factors (ethnicity, family history of T2DM, signs of insulin resistance or conditions associated with, maternal gestational diabetes) [13, 55]. No guidelines recommend universal screening in children and adolescents.

#### Screening for smoking

Smoking is highly addictive and a major risk factor for CVD [56]. Worldwide, it kills over 5 million persons per year [56]. Helping smoking cessation is efficient in adults and the USPSTF recommends that clinicians ask all adults about tobacco use and provide tobacco cessation interventions for those who use tobacco products [57]. As most adult smokers started to smoke during late childhood and adolescence [58], interventions to prevent initiation or help cessation of smoking during childhood and adolescence are warranted. Cochrane reviews have shown that school- and family-based interventions can help prevent smoking initiation in children and adolescents [59, 60].

Evidence on the effect of interventions for smoking cessation in adolescents is less convincing [58, 61, 62]. The American Medical Association’s (AMA) Guidelines for Adolescent Preventive Services (GAPS) recommend that “all adolescents should be asked annually about their use of tobacco products including cigarettes and smokeless tobacco” [61]. However, a recent systematic review assessing evidence for the efficacy and harms of primary-care interventions to reduce tobaccos use in children and adolescents has shown that smoking initiation could be prevented by behavior based interventions but that neither behavior-based nor pharmacologic interventions improved cessation rate [62]. Based on this review, the USPSTF recommends that primary care clinicians provide interventions, including education or brief counseling, to prevent initiation of tobacco use among school-aged children and adolescents [63]. The USPSTF does not recommend screening for smoking.

## Conclusion

Since 50 years, the prevention of CVD is a success story among adults of high-income countries. Indeed, CVD mortality rates have sharply decreased, e.g., in the United-States, Canada or Switzerland, in both sexes and in all age strata of the population [64]. Population-based as well as high-risk prevention strategies have contributed to this large decrease in CVD [65]. It is increasingly accepted that CVD prevention strategies should include children as well as adults [66]. Nevertheless, even if the pathogenic process of CVD originates in childhood, it does not imply that the model of adult CVD clinical prevention strategies is applicable to children. Our review suggests indeed that there is no compelling evidence to recommend universal screening for elevated blood pressure, abnormal blood lipids, abnormal blood glucose, or smoking in children and adolescents. Targeted screening of these risk factors could be useful but specific screening strategies have to be evaluated [44, 45, 67].

Research is needed to identify target populations, screening frequency, intervention and follow-up. Further, research is needed to assess the effect of early life screening and treatment of CVD risk factors. Since CVD are remote outcomes, rarely occurring before the age of 60 years, the effect of early life screening and treatment should be tested in trials using more proximal outcomes, i.e., proxies such as carotid intima-media thickness which have been shown to be associated with CVD risk factors in childhood and adolescence. Meanwhile, efforts should rather focus on the primordial prevention of CVD risk factors [12] and at maintaining a lifelong ideal cardiovascular health through environmental, policy, and educational approaches [68].

## Abbreviation

CVD: Cardiovascular diseases
BP: Blood pressure
USPSTF: U.S. Preventive Services Task Force
NHLBI: U.S. National Heart Lung, and Blood Institute
